# Seven different genes encode a diverse mixture of isoforms of Bet v 1, the major birch pollen allergen

**DOI:** 10.1186/1471-2164-7-168

**Published:** 2006-07-04

**Authors:** Martijn F Schenk, Ludovicus JWJ Gilissen, Gerhard D Esselink, Marinus JM Smulders

**Affiliations:** 1 Allergy Consortium Wageningen, Wageningen University, Wageningen, The Netherlands; 2 Plant Research International, Wageningen University, Wageningen, The Netherlands

## Abstract

**Background:**

Pollen of the European white birch (*Betula pendula*, *syn. B. verrucosa*) is an important cause of hay fever. The main allergen is Bet v 1, member of the pathogenesis-related class 10 (PR-10) multigene family. To establish the number of PR-10/Bet v 1 genes and the isoform diversity within a single tree, PCR amplification, cloning and sequencing of PR-10 genes was performed on two diploid *B. pendula *cultivars and one interspecific tetraploid *Betula *hybrid. Sequences were attributed to putative genes based on sequence identity and intron length. Information on transcription was derived by comparison with homologous cDNA sequences available in GenBank/EMBL/DDJB. PCR-cloning of multigene families is accompanied by a high risk for the occurrence of PCR recombination artifacts. We screened for and excluded these artifacts, and also detected putative artifact sequences among database sequences.

**Results:**

Forty-four different *PR-10 *sequences were recovered from *B. pendula *and assigned to thirteen putative genes. Sequence homology suggests that three genes were transcribed in somatic tissue and seven genes in pollen. The transcription of three other genes remains unknown. In total, fourteen different Bet v 1-type isoforms were identified in the three cultivars, of which nine isoforms were entirely new. Isoforms with high and low IgE-reactivity are encoded by different genes and one birch pollen grain has the genetic background to produce a mixture of isoforms with varying IgE-reactivity. Allergen diversity is even higher in the interspecific tetraploid hybrid, consistent with the presence of two genomes.

**Conclusion:**

Isoforms of the major birch allergen Bet v 1 are encoded by multiple genes, and we propose to name them accordingly. The present characterization of the Bet v 1 genes provides a framework for the screening of specific *Bet v *1 genes among other *B. pendula *cultivars or *Betula *species, and for future breeding for trees with a reduced allergenicity. Investigations towards sensitization and immunotherapy should anticipate that patients are exposed to a mixture of Bet v 1 isoforms of different IgE-reactivity, even if pollen originates from a single birch tree.

## Background

Pathogenesis-related class 10 (PR-10) proteins constitute the largest group of aeroallergens and are among the four most common food allergens [1]. The main allergen is a PR-10 pollen protein from the European white birch (*Betula pendula*) termed Bet v 1 [2]. Birch pollen is a major cause of Type I allergies in the temperate climate zone of the northern hemisphere. Over 95% of the tree pollen-sensitized patients in Scandinavia display IgE binding to Bet v 1, while 60% react exclusively to this allergen [3]. Pollen of other Fagales species contains Bet v 1 homologues that share epitopes with Bet v 1 [4].

PR-10 proteins are present as a multigene family across a range of phylogenetically distant species, including Gymnosperms, Monocots, and Dicots [5-7]. As a consequence, several foods contain Bet v 1 homologues, including nuts, vegetables, and Rosaceae fruits [6,8,9]. Patients that are sensitized to Bet v 1 may experience mild allergic symptoms upon consumption of these foods due to IgE cross-reactivity. Symptoms of this so called oral allergy syndrome (OAS) are mainly limited to the oral cavity. Cross-reactivity has clearly been demonstrated by allergic responses to the celery protein Api g 1, which is initiated by sensitization to Bet v 1 [8].

The *B. pendula *genome contains multiple *PR-10 *genes with varying expression patterns. Among these, the Bet v 1 allergens are expressed in pollen. The first Bet v 1 isoform was identified by immunoscreening a pollen cDNA expression library with serum of birch pollen allergic patients [2]. Other Bet v 1 isoforms have been sequenced by various authors since then [10-13]. Pollen mixtures from multiple trees were found to contain multiple Bet v 1 isoforms [13]. Bet v 1 isoforms differ in the ability to bind IgE and in the T-cell proliferation score [14]. Two other types of PR-10 proteins were detected in birch cells that were grown in a liquid medium in the presence of microbial pathogens [15]. These proteins are expressed in roots and leaves under basal conditions or induced under various stress-related conditions [15,16]. The *PR-10 *genes from *B. pendula *form a homogeneous group, based on sequence similarities. Homogeneity is suggested to be maintained by concerted evolution [17]. Arrangements of *PR-10 *genes into clusters, such as found for Mal d 1 genes in apple (*Malus domestica*), may facilitate concerted evolution [18].

Given the prominent role of Bet v 1 allergens in the sensitization to hay fever and OAS, birch is a relevant target for development of allergy prevention strategies. Selection and breeding of hypoallergenic trees or the application of genetic modification to develop these may potentially reduce the allergenic load caused by birch. Knowledge on the diversity of *PR-10 *genes, their expression, and allergenicity of the gene products is required to facilitate these strategies. In the present study, we amplified, cloned and sequenced *PR-10 *alleles from three *B. pendula *cultivars to establish the number of *PR-10/Bet v 1 *genes and the isoform diversity within a single tree.

## Results

### PCR recombination artifacts

When PCR amplification is performed on groups of closely related sequences, such as the PR-10 gene family, accurate sequences are essential to distinguish between members. When we initially determined the relationships among the recovered sequences, most clades in the Bayesian consensus tree had low posterior probabilities. Several sequences occupied intermediate positions between well-defined clusters. This suggested the possibility of recombination. Recombination could have occurred *in vivo *through a crossing-over or gene conversion between prior existing genes, or *in vitro *during the PCR through strand-switching or re-annealing of incompletely amplified fragments.

**Figure 1:**
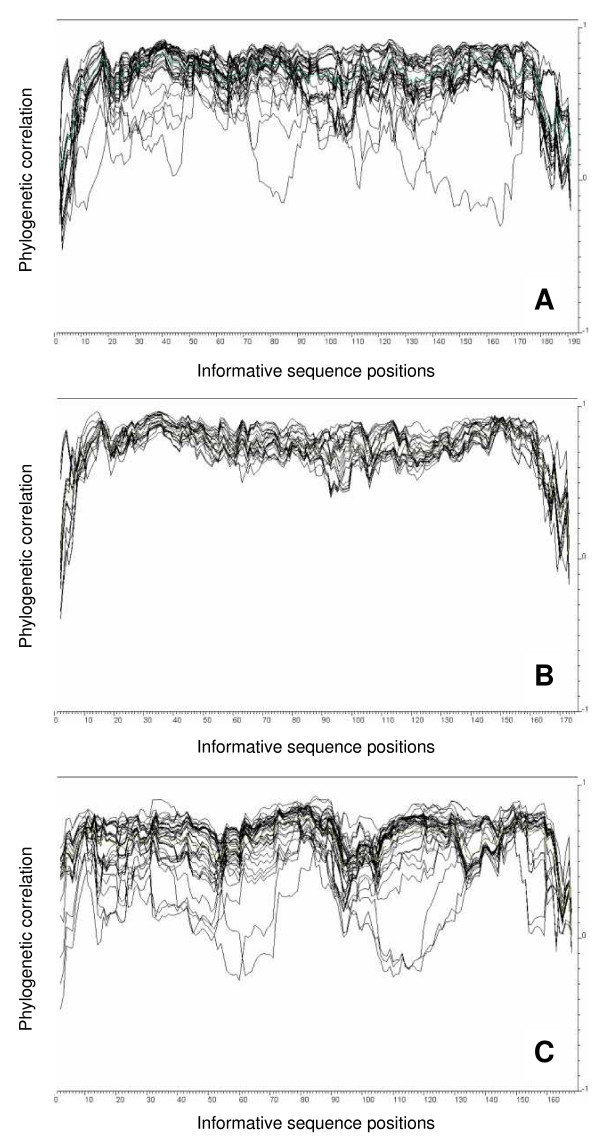
**Phylogenetic profiles for detection of recombination**. Phylogenetic profile of the sequences from *B. pendula *'Long Trunk' obtained after a PCR of (**a**) 30 cycles (n = 72 sequences) and (**b**) 20 cycles (n = 53). (**c**) Phylogenetic profile of the GenBank PR-10 sequences from *B. pendula *(n = 66). The x-axis represents the sequence position (5'-3' including only informative positions). The y-axis indicates the phylogenetic correlation. Low values are indicative for recombination [27]. Low values at the edges are artifacts of the employed method.

Evidence supports the view that recombinant sequences were PCR artifacts. Recombination signals were abundant in the sequences obtained after the 30-cycle PCR (Figure 1a) and virtually absent when 22 cycles were employed (Figure 1b). Several GenBank sequences showed clear evidence for recombination too (Figure 1c). Putative recombinants from our experiments lacked or nearly lacked unique mutations and could be separated into two or three stretches that were identical to other sequences obtained. The GenBank recombinants shared close to 100% sequence identity to combinations of other accessions.

The use of independent PCRs resolves which sequences are genuine, since the probability of isolating identical artifacts in independent PCRs is extremely low. Most sequences without recombination signal were confirmed in independent PCRs (Table 1), while those with a recombination signal were not. The only exception was the *PR-10.03B02.01 *allele from 'Tristis' that was found in three independent PCRs. This allele was an *in vivo *recombination of the first 300 bp, including the intron, from the *PR-10.03D *gene and 183 bp from the original *PR-10.03B *gene as found in 'Schneverdinger Goldbirke' and 'Long Trunk'. Putative recombination artifacts were quite abundant in the 26 and 30-cycle PCR (27–46% of the sequences), but rare in the 22 and 24-cycle PCR (2–11%) (Table 1). We conservatively maintained the sequences that were confirmed in independent PCRs and those with at least three unique mutations for further analysis. A minimum of three unique mutations was chosen to ensure that potential base mis-incorporation artifacts were excluded.

Several of the *PR-10 *sequences from *B. pendula *that were available from GenBank/EMBL/DDBJ also showed clear evidence for recombination (Figure 1c). However, it was not possible to do a similar check as mentioned above for the GenBank sequences, and we can only hypothesize on the presence of artifact sequences without such direct evidence. Given the regular occurrence of artifact sequences in our experiments, we maintained only those 40 GenBank sequences that were not under suspicion of recombination artifacts for further analysis.

### Phylogenetic analysis: newly isolated sequences

We sequenced 404 individual clones in both directions (Table 1). Fourteen different sequences were identified in the diploid cultivar 'Schneverdinger Goldbirke', 7 of which were unique for this cultivar. Fifteen different sequences (of which 10 were unique) were identified in the diploid cultivar 'Tristis'. Approximately twice as many different sequences, namely 28, were identified in the tetraploid cultivar 'Long Trunk'; of these, 16 sequences were of *B. pendula *origin (8 unique), and 12 sequences were from another *Betula *species (all unique). In total, 32 different sequences were found.

The Open Reading Frame (ORF) of the sequences was highly conserved and the alignment was straightforward. All but one ORF contained 483 nucleotides, coding for a putative protein that is 160 amino acids long. One sequence from 'Schneverdinger Goldbirke' required the inclusion of an indel between base 388 and 389 of the consensus. This sequence was denoted as a pseudogene, since the indel introduced a stop codon at 7 codons downstream. It cannot be excluded that this pseudogene is expressed, since the stop codon was located near the 3' end. The intron position was identical in all sequences and located at codon 62, being inserted between the first and second nucleotide. Most alleles had 5' splicing sites of AG:GT, with the exception of one allele that had a GG:GT splicing site. The 3' splicing sites were AG:GC or AG:GA. This is in concordance with known motifs for plant introns. The introns were relatively AT-rich (55–65%).

**Figure 2:**
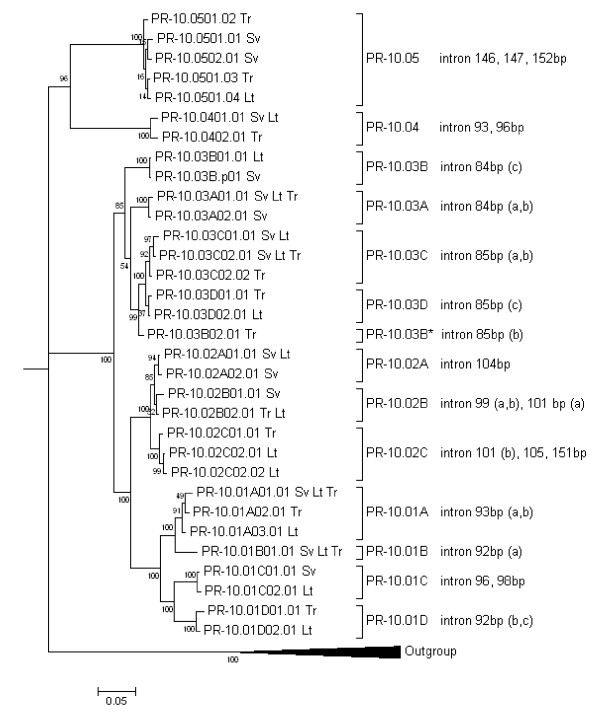
**Bayesian phylogenetic tree of the *PR-10 *sequences from *B. pendula***. Bayesian phylogenetic tree of the *PR-10 *sequences from *B. pendula *'Schneverdinger Goldbirke' (Sv), 'Tristis' (Tr), and the *B. pendula *alleles from 'Long Trunk' (Lt). The 'Long Trunk' alleles that belong to the unknown parental species are not included in this figure. Numbers on the branches represent posterior probabilities after running a Markov chain Monte Carlo search for 1,000,000 generations. Sequences of *PR-10 *genes from *Malus domestica *(apple, X83672, Z72425, Z72427), *Prunus armeniaca *(apricot, AF020784), *P. avium *(cherry, U66076), and *Pyrus communis *(pear, AF057030) were used as outgroup. Each cluster that is identified as a putative gene has maximally two alleles per cultivar. Genes are classified into five major groups. The intron length is indicated on the right. If multiple introns of the same length exist within one group, the different types are shown between brackets. ^*1 ^PR-10.03B02.01 from 'Tristis' was an *in vivo *recombination of the *PR-10.03D *gene and the original *PR-10.03B *gene.

We determined the relationships among the *PR-10 *sequences from *B. pendula*. For this, the 'Long Trunk' sequences that were designated to the other parental species were excluded. Excluding primer traces, 171 of the 452 aligned exon positions were variable, while 150 positions were phylogenetically informative. The consensus tree from the Bayesian analysis indicated several well-defined clusters (Figure 2). We implemented a cut-off level of 98% identity and allowed maximally two alleles per cultivar per gene to estimate the number of genes. In this way, we putatively identified ten genes in 'Schneverdinger Goldbirke', eleven in 'Tristis', and thirteen in 'Long Trunk'. Thirteen different genes were distinguished when the information was combined. Each gene was identified as such in at least two birch cultivars and was characterized by a distinct intron, in most cases of a different size between 84 and 152 bp (Figure 2). The similarity between different alleles of one gene ranged from 98.9 to 100% identity in the exons, which corresponds to 0–5 SNPs. *Bet v 1.02A *and *Bet v 1.02B *were not well distinguished in the coding sequences, but had distinguishable introns. The pseudogene *PR-10.03B-p01 *from 'Schneverdinger Goldbirke' was identical to the *PR-10.03B *allele from 'Long Trunk' except for its indel.

When all alleles from 'Long Trunk' were included, 182 variable positions were identified among the 452 aligned exon positions. 154 positions were phylogenetically informative. The topology of the consensus tree from the Bayesian analysis showed that seven specific 'Long Trunk' genes were clustered pair wise to the *B. pendula *genes and these are likely to represent orthologuous genes from the second *Betula *species (not shown). Given the high identity (up to 100%) to PR-10 sequences from *B. ermanii*, this species, or a close relative, is likely to represent the second parental species (unpublished data, Schenk *et al*.). Intron sequences of orthologuous genes mostly showed slight differences in length or base pair composition. Three genes were recovered only from the unknown parent species and five only from *B. pendula*, indicating that *Betula *species do not necessarily have the same (number of) genes.

### Phylogenetic analysis: GenBank sequences versus newly obtained sequences

All but two GenBank sequences had an ORF of 483 base pairs that coded for 160 amino acids and was generally interrupted by a position-conserved intron. Two sequences required the inclusion of an indel, following base 354 of the consensus sequence. This resulted in a stop codon directly after the insertion. The GenBank dataset was combined with the newly obtained sequences. Primer traces were discarded, resulting in an aligned stretch of 425 bp from base 28 to 452 of the consensus. Several GenBank sequences are identical at this stretch, reducing the number of unique GenBank sequences in the analysis to thirty-three. 173 out of 425 aligned positions were variable, while 146 were phylogenetically informative. The information from the Bayesian consensus tree was added to Table 2 by indicating in which gene cluster the GenBank alleles landed. Similarly to the analysis of the newly isolated sequences we used a cut-off of 98% identity. In the resulting classification, 35 out of 40 alleles that were assigned to a particular gene showed more than 99% identity for the exons. The lowest similarity of an allele that still clustered with a particular gene in the phylogenetic analysis was 98.4% identity with the reference sequence (Table 2).

We classified the genes into five subfamilies (I–V) based on identities of the coding regions and the intron. The average identity between alleles within each subfamily was 95–100%. The GenBank sequences were in part derived from RNA extractions from specific tissues. We used this tissue information to predict the transcription of the *PR-10 *genes (Table 2). For this, alleles of a single gene are assumed to have the same mode and location of transcription. Subfamily I and II consist of respectively four and three genes and include alleles that are homologous to the pollen-expressed Bet v 1 allergens [2,13]. For four genes within these subfamilies, we found alleles that were 100% identical to pollen mRNA-derived sequences previously deposited into GenBank. Alleles from two other genes were 99.8% identical (1 SNP difference) to pollen mRNA-derived sequences from GenBank, which we take to predict the location of transcription for these six genes with a very high level of confidence. For only one of the genes in subfamily II, there was no mRNA-derived homologue in the GenBank database, but the high homology (97.8–98.2%) to the other genes in this subfamily suggests that this gene will be expressed in pollen as well.

Subfamily III consists of four genes, two of which have alleles that are 99.5% identical to homologues of the previously described *ypr10a *and *ypr10b *sequences, which are transcribed in roots and leaves [15]. Transcription of the other two genes in subfamily III is unknown, as is the transcription of the single gene in subfamily IV. Subfamily V consists of a single gene. One of the recovered alleles was 100% identical to the previously described *ypr10c *sequence that is also expressed in roots and leaves [15]. Given the fact that all sequences in subfamily III-V are less than 90% homologous to pollen-derived mRNAs and that there is no evidence of expression in pollen, we consider these as non-allergens.

### Nomenclature

All recovered *B. pendula *alleles were added to Table 2. This table also lists all known GenBank accessions of Bet v 1 sequences that were not under suspicion of artifacts. If sequences were previously named, we included a cross-reference to the original nomenclature (Bet v 1a-n) as well as to the nomenclature of the allergen nomenclature committee (Bet v 1.0101 to 1.3101) [19]. Gao *et al*. [18,20] designed a nomenclature system for the Mal d 1 to Mal d 4 allergens that differentiates between alleles from different genes. We have adopted their system in Table 2. Protein sequences that have less than 95% identity are designated according to the current allergen nomenclature. A Latin letter is added to the iso-allergen name for those genes that have more than 95% identity. Two numerals are added for each allelic variant at the protein level. Two additional numerals indicate silent mutations. For example, *Bet v 1.02C02.02 *refers to a silent mutation in the second protein variant of the *Bet v 1.02C *gene, which shares more than 95% identity with *Bet v 1.02A *and *B*. The *PR-10 *sequences that were not allergens (not pollen-expressed) are named in a similar fashion, except that names started with *PR-10 *instead of *Bet v 1*. If future evidence would indicate that a particular PR-10 gene is expressed in pollen after all, the name can easily be modified by replacing the PR-10 tag with Bet v 1.

### PR-10 proteins and allergenicity

**Figure 3:**
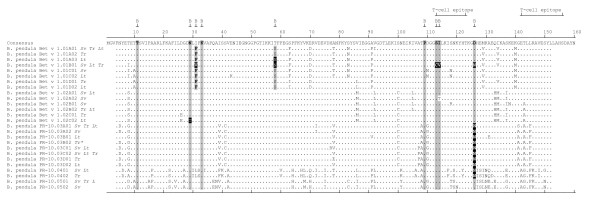
**Amino acid sequences, amino acids that affect IgE-reactivity, and T-cell epitopes of the PR-10 proteins**. Amino acid sequences of the PR-10 proteins from *B. pendula *'Tristis' (Tr), 'Schneverdinger Goldbirke' (Sv), and the *B. pendula *alleles from 'Long Trunk' (Lt). Amino acids associated with high allergenicity are marked with grey boxes and those associated with low IgE-reactivity (located within B-cell epitopes) are marked with black boxes [12, 14]. The locations of the two major T-cell activating regions are indicated above the consensus [22].

A high similarity between proteins increases the chance that they share epitopes, while on the other hand, a single amino acid change may influence allergenicity drastically. The high homogeneity among the PR-10 genes of *B. pendula *was reflected by a higher allelic variation at the nucleic acid level compared to the protein level. Hence, the 45 different genomic sequences encoded 32 different putative isoforms (Figure 3). This is consistent with the relatively large number of synonymous mutations compared to the number of non-synonymous mutations (K_a_/K_s _ratios) [21]. The number of synonymous (K_s_) and non-synonymous (K_a_) substitutions per site were calculated from pair wise comparisons of all alleles from the three *B. pendula *varieties. The average value was 0.080 for K_a _and 0.247 for K_s_, resulting in an average K_a_/K_s _ratio of 0.33 (n = 45). Analysis of the occurrence of non-synonymous mutations per codon indicated two interesting regions. The region between codon Asn^42 ^and Ile^56 ^lacked non-synonymous mutations and is characterized by a phosphate-binding loop with the sequence motive GxGGxGx (Figure 3). Relatively many amino-acid differences were present beyond codon 125, especially between isoforms of the genes *PR-10.04/PR-10.05 *and *PR-10.01 *to *PR-10.03 *(Figure 3).

Previous research has identified isoforms with varying IgE reactivity within mixtures of pollen. In fact, this is also true for pollen from a single tree. Ferreira *et al*. [14] made a distinction between isoforms with high, intermediate and low IgE-binding activity. The high IgE-binding isoform Bet v 1a (X15877) clustered with alleles of gene *Bet v 1.01A *and was 100% identical at the protein level to the Bet v 1.01A01 isoform. The intermediate IgE binding isoforms Bet v 1c and f (X77265, X77268) clustered with alleles of the genes *Bet v 1.01C *and *Bet v 1.02C *and differed by one amino acid from the Bet v 1.01C01 and the Bet v 1.02C02 isoforms. The low IgE binding isoform Bet v 1d (X77266) clustered with alleles of gene *Bet v 1.01B*, and was 100% identical at the protein level to the Bet v 1.01B01 isoform. Nine amino acids have been identified that affect the allergenicity of the Bet v 1 proteins on the B-cell level [12,14]. These are marked in Figure 3. The tetraploid 'Long Trunk' contained several isoforms with unique amino acid substitutions due to its putative hybrid origin. This greatly enlarges the variation in putative IgE-reactivity among isoforms of this cultivar.

Two major T-cell binding epitopes have been identified, which are positioned between amino acids 112–123 and 142–156 [22]. Activation of T lymphocytes with the Bet v 1_142–156 _epitope induced cytokine production (IL-4, IL-5). The major T-cell epitope Bet v 1_142–156 _has an amino acid variation at position Thr^143^. At this position the genes *Bet v 1.02B *and *Bet v 1.02C *code for Ala^143 ^instead. We identified an additional variation in the *Bet v 1.01D *gene from the tetraploid 'Long Trunk', namely the presence of Arg^150 ^instead of Ser^150^. The T-cell epitope Bet v 1_112–123 _is more variable and contains amino acid variations in the genes *Bet v 1.01B*, *Bet v 1.01C*, *Bet v1.02C *and *Bet v 1.02D *(Figure 3).

## Discussion

### PCR artifacts

When PCR amplification is performed on groups of closely related sequences, such as the PR-10 gene family, accurate sequences are essential to distinguish between members. We used *Pfu *polymerase, which has proofreading functionality and reduces the number of base substitution error rates. However, previous research has shown that *Pfu *polymerase may generate more and more complex recombination artifacts than *Taq *polymerase [23,24] through incomplete primer extension and re-annealing to a different template [25], or strand switching between different templates [26]. Reducing the number of cycles [24] was an efficient solution to lower the amount of artifacts.

We identified PCR recombination artifacts in several of our sequences using the computer program Phylpro [27]. The comparison of independent PCRs enabled sequence validation and exclusion of both recombination and base substitution artifacts. The high occurrence of recombination artifacts in our experiments (27–46% after 30 PCR cycles) is not uncommon. For example, Wang and Wang [28] report 32% recombination artifacts after 30 cycles of PCR amplification for 16S rRNA genes. The occurrence of recombinant sequences within a mixture of approximately 15 different sequences is expected to be high, because almost all recombinations are detected. Half of the recombinations would, for example, remain undetected if only two templates are present. The presence of 13 genes also increases the amount of template compared to a single copy gene.

Using the same analysis to detect recombination in GenBank/EMBL/DDJB accessions, we observed a recombination signal in 22 out of 62 (35%) accessions. These are not necessarily all PCR artifacts, as some recombinations may have occurred *in vivo *during evolution of the genes. However, we found only one true recombinant in our dataset and no evidence of past recombination events in the comparison between sequences from two different species (within the hybrid). This indicates that the occurrence of *in vivo *recombination is probably rare.

Base substitution error rates for mixtures of non-proofreading *Taq *and proofreading *Pfu *are approximately 5.6 × 10^-6 ^under optimal conditions [29]. This error rate is ~2–4 fold higher when only *Taq *is used [29,30]. Without confirmation in independent PCRs these errors can not be excluded and it is very likely that Bet v 1 sequences with base substitution errors have been deposited into GenBank/EMBL/DDJB. Therefore, not all published isoforms will be clinically relevant. In addition, the clinical relevance of the isoforms will be influenced by their expression levels. If multiple allergen isoforms exist, there is a risk for selecting a recombinant isoform with low IgE-reactivity as a diagnostic tool, or even selecting isoforms that resulted from PCR artefacts. We therefore strongly suggest the use of primers that are highly specific for one gene [18] or, preferentially, the application of multiple independent PCRs to facilitate sequence validation in future sequencing work on allergens.

### PR-10 and Bet v 1 genes

The PR-10 gene family of *Betula pendula *was shown to encompass at least thirteen genes. This is a conservative estimate since we used strict inclusion criteria. The distinction between genes is supported by the presence of a distinct intron. Each gene was identified as such in at least two birch cultivars. We attributed previously described GenBank sequences to these genes. Alleles from ten identified genes had previously been described [2,10-13], while we identified three new genes. The genes are grouped into five subfamilies, based on sequence homologies in the ORF and intron. Differences in transcription coincide with the division between subfamilies. An organization of *PR-10 *genes into subfamilies was also reported for *Malus domestica *[18] and for *Pinus monticola *[5].

A striking feature of the *PR-10 *isoforms in *B. pendula *is their homogeneity, which may extend to other Fagales species, such as alder (*Alnus glutinosa*) and hazel (*Corylus avellana*). The intron has conserved 3' and 5' splicing sites and is always located at codon 62, as is *e.g*. reported for *C. avellana *and *M. domestica *[11]. High homogeneity may result from strong purifying selection or from concerted evolution. The presence of low K_a_/K_s _ratios among the isoforms suggests the occurrence of purifying selection. Evidence for concerted evolution is present in the overall gene tree of the PR-10 family [17]. Concerted evolution causes genes to evolve as a single unit with members exchanging genetic information through gene conversion and unequal crossing-over. Tandemly arranged genes may have high conversion rates [31], while this is a prerequisite for the occurrence of unequal crossing-over. Most *PR-10 *genes in apple map to two loci and are arranged in a duplicated cluster [18]. This organization may be a common feature for PR-10 genes. However, as pointed out by Nei and Rooney [32], the molecular mechanism of gene conversion is not well understood, and the model of birth-and-death evolution of genes may also explain the evolution of the PR-10 gene family. The presence of pseudogenes, although at a low frequency, is therefore of particular interest. An analysis of a species at an intermediate evolutionary distance, such as *C. avellana*, would be useful to clarify which mechanisms determine the evolution of PR-10 genes and to investigate a possible recent radiation of PR-10 genes.

The birch genome contains at least seven pollen-expressed genes that encode a mixture of Bet v 1 isoforms with varying IgE-reactivity. Swoboda *et al*. [13] found that pollen mixtures from multiple trees contain multiple Bet v 1 isoforms. We identified 14 different Bet v 1 isoforms in the three cultivars, nine of which are entirely new. The IgE-reactivity has been tested for several isoforms using recombinant proteins [14]. The allergenicity of the new isoforms can be examined in the future by expressing the isoforms as recombinant proteins and use these in a SPT or T-cell activation tests. Ferreira *et al*. [14] divided the Bet v 1 isoforms into three groups according to their IgE-reactivity and confirmed the division between high, moderate, or low IgE-reactivity in a Skin Prick Test (SPT). One high and one low IgE-reactive isoform from their analysis were 100% identical to isoforms that we have obtained from a single tree, while two intermediate IgE-reactive isoforms differed only by one amino acid from the alleles of two other identified genes. This strongly suggests that isoforms of different IgE-reactivity are in fact alleles encoded by different genes. Thus, each examined cultivar has the genetic background to express a mixture of isoforms with a high, moderate, and low IgE-reactivity. We plan to confirm this at the protein level in the near future.

### Nomenclature

The nomenclature of Bet v 1 raises several issues. The first isoforms were termed Bet v 1a to Bet v 1n by Swoboda *et al*.[13], but these have subsequently been renamed and incorporated into the official database of the allergen nomenclature committee [19]. This database currently lists 37 allergen isoforms that have been termed Bet v 1.0101 to 1.3101. However, as can be seen from the list of known isoforms in Table 2, not all published isoforms have been added to this list, even though several of these isoforms were obtained from pollen mRNA. On the other hand, isoforms which have been recovered only from mRNA from roots and leaves are included as allergens (Bet v 1.1101 to Bet v 1.1301). Also, several of the described isoforms are highly suspicious as we observed clear recombination signals. As a result, the list is a random series of alleles that belong to different genes and has no biological basis. Similar problems were described for the Mal d 1 and Mal d 3 allergens by Gao *et al*. [18,20]. To allow differentiation between alleles from different genes for the Bet v 1 alleles we have adopted their system.

### Allergenicity of birch trees

The exact isoform composition of the three cultivars differed due to allelic variations. This may result in differences in allergenicity between cultivars. However, an exact copy of the most allergenic allele, *Bet v 1a*, was present in all three cultivars. Quantity measurements on expression indicate that Bet v 1a is the dominant isoform in pollen [13]. Given that the diversity of *Bet v 1 *isoforms within a single tree is larger than the diversity between the examined *B. pendula *cultivars, a characterization of *Bet v 1*-type isoforms should be done in other *Betula *species as well. Investigations towards sensitization and immunotherapy should anticipate that patients are exposed to a mixture of Bet v 1 isoforms of different IgE-reactivity, even if pollen originates from a single birch tree. Differences in allergenicity between birch trees may also result from variation in allergen content. Variation in allergen content has *e.g*. been shown for apple [33] and olive pollen [34].

Many Betula species and Betula hybrids have higher ploidy levels (tetraploid, hexaploid, and even octaploid) than *B. pendula *and are likely to contain increased numbers of allergen isoforms, as we found in the tetraploid cultivar 'Long Trunk'. For example, the tetraploid *B. pubescens *is dispersed throughout Europe, while other exotic birch species are increasingly introduced as cultivars, contributing to a larger allergen pool. However, interspecific *Betula *hybrids, which have a higher ploidy level, may also pose a potential source of hypoallergenic trees. Especially hybrids between less related species often display a reduced fertility, which may result in a reduced or aborted pollen production.

If breeding for hypoallergenic trees is implemented, approaches should take into account that the Bet v 1/PR-10 genes may be clustered and differences in allergenicity between clusters of genes may be used to guide breeding efforts. Clustering may be determined by mapping studies [18] and by screening and partial sequencing of a genomic library. In the genome sequence of *Populus *[35] we indeed can observe an organization of PR-10 genes into clusters. Other approaches to generate hypoallergenic trees may include the search for sterile or low pollen producing trees, or the application of RNA interference technology, which proved to be successful to silence the Mal d 1 allergens in apple [36] without phenotypic abnormalities. One issue that needs the be resolved is that although birch PR-10/Bet v 1 proteins have been suggested to act as plant steroid carriers [37], the exact conditions under which transcription is induced are still unknown. Given the abundance of Bet v 1 in birch pollen, silencing may affect pollen viability. However, for breeding of hypoallergenic cultivars that are propagated vegetatively this would be considered a welcome side-effect.

## Conclusion

We have shown that the PR-10 gene family of *Betula pendula *encompasses at least thirteen genes that can be grouped into five distinct subfamilies. Differences in expression coincide with the division between subfamilies. Genes from two subfamilies were shown to be transcribed in pollen, based on a high (99.8–100%) homology with cDNA sequences available in GenBank/EMBL/DDJB. The seven genes that belong to these subfamilies encode a mixture of Bet v 1 isoforms of varying IgE-reactivity. The present characterization of the PR-10 family in birch provides a framework for the screening of *Bet v *1 genes among other *Betula *species or *B. pendula *cultivars and for potential breeding approaches for birch trees with a reduced allergenicity.

## Methods

### Plant material

The natural distribution range of *B. pendula *Roth (*syn. B. verrucosa*) covers almost the whole of Europe. Several *B. pendula *cultivars have been bred, including interspecific hybrids (also referred to as interspecies hybrids) between *B. pendula *and other *Betula *species. We collected young leaves from three *B. pendula *cultivars in the collection of PPO Boskoop (WUR, the Netherlands), namely 'Long Trunk', 'Schneverdinger Goldbirke', and 'Tristis'. Fresh leaf samples were sent to Plant Cytometry Services (Schijndel, The Netherlands) and screened by flow cytometry to estimate the ploidy level. Diploid (*B. pendula*) and tetraploid (*B. pubescens*) controls were included. The cultivars 'Schneverdinger Goldbirke' and 'Tristis' were diploid, while the cultivar 'Long Trunk' was tetraploid. The latter is likely to be an interspecific hybrid between *B. pendula *and a second, unknown, *Betula *species. The alleles recovered from 'Long Trunk' were either assigned to *B. pendula *based on sequence and intron similarity or were considered specific for this cultivar. The specific 'Long Trunk' alleles were analyzed separately. DNA was extracted using the DNeasy Plant Mini kit (Qiagen) according to the manufacturer's instructions.

### PCR, cloning, and sequencing

*PR-10 *alleles were amplified from birch DNA with primers designed after two cDNA sequences (X15877, X77601). The primers were complementary to the regions around the start and stop codons (shown in bold); BpI-For: 5'-AATCTCTCAGGCCATC**ATG**GGTG-3', BpI-Rev: 5'-**TA**GTTGTAGGCATCGGAGTGTGC-3', BpII-For: 5'-ATCTCAGGTGATCATC**ATG**GGTG-3', and BpII-Rev: 5'-**TA**GTTGTAGGCATTTGGGTGTGC-3'.

PCR amplification with both primer pairs was performed with mixtures consisting of 2 μl dNTP (1 mM), 2 μl 10× Reaction buffer, 0.8 μl MgCl_2 _(25 mM), 1.2 μl forward primer (10 pmol/μl), 1.2 μl reverse primer (10 pmol/μl), 0.11 μl of a 1:9 mixture of *Pfu *polymerase (Stratagene) and *Taq *polymerase (Goldstar)(5U/μl), and 20–80 ng template DNA. H_2_O was added to obtain a total volume of 20 μl. PCR mixtures were subjected to the following conditions: initial heating step at 95°C for 15 minutes, denaturation at 94°C for 30 s, annealing at 50°C for 45 s, and extension at 72°C for 60s. A final extension step of 10 min at 72°C was added after 22–30 cycles. Given the observation of recombination among the recovered sequences, we subsequently varied the number of PCR cycles at intervals of 2 cycles. The minimum number of cycles was established by visual inspection of the amplification products on agarose gel at 22 for the BpI primer pair and at 24 for the BpII primer pair. Originally, 30 PCR cycles had been used. We repeated the experiment at 22–24 cycles to ensure that amplification was in its linear phase.

To obtain the A-tailing that facilitates the ligation procedure, five additional cycles were run on 1–4 μl of PCR product with *Taq *polymerase (Goldstar). PCR conditions were similar as described above. PCR products were purified with the MinElute PCR Purification Kit (Qiagen). Purified samples were ligated into the pGEM-T easy Vector (Promega) and established in *Escherichia coli *XL1 Blue competent cells (Stratagene) according to the manufacturer's instructions. White colonies were picked from agar plates and grown overnight at 37°C in freeze medium. PCR-based screening was performed with vector-specific M13 primers. PCR products were purified with Sephadex G-50 (Millipore). The DYEnamic™ ET Terminator Cycle Sequencing Kit (Amersham) was used for the sequence reaction. Sequence products were analyzed on a 96-capillary system (ABI 3730xl).

Genomic *B. pendula *sequences have been submitted to GenBank as DQ296566–DQ296598 and DQ325525–DQ325535, and the specific 'Long Trunk' sequences as DQ296599–DQ296610.

### GenBank sequences

Sixty-six PR-10 sequences were obtained from GenBank/EMBL/DDJB by searching with MegaBLAST for entries from *B. pendula *with more than 60% sequence identity to Bet v 1a (X15877). The search results included nineteen genomic DNA (gDNA) sequences: Z72429–Z72438[11]; AJ001551–AJ001557 (Cvitanich and Larsen, direct submission); AJ289770 and AJ289771 (Pellinen *et al*, direct submission). The accessions Z72435-8 were singletons with a relatively low homology to our and other GenBank *Betula *sequences and were excluded from the analysis. Forty-seven cDNA sequences were found: X15877[2]; X77200, X77265–X77274, X81972, X82028[13]; X77599–X77601[15]; Z80098–Z80106 (Larsen, direct submission); AF124837–AF124839[12]; AJ002106–AJ002110, and AJ006903–AJ006915[10].

### Phylogenetic analysis

Nucleotide sequences were aligned using CLUSTALW [38]. Bayesian phylogenetic analysis was performed with MrBayes 3.1.1 [39]. The maximum likelihood model employed 6 substitution types, with base frequencies set to the empirically observed values. Rate variation across sites was modeled using a gamma distribution. The Markov chain Monte Carlo search was run twice with 4 chains for 1,000,000 generations. Topology and model parameters were sampled every 100th generation and used to estimate model parameters and to determine the posterior probabilities of clades. The first 100,000 generations were discarded as "burn in". The outgroup was composed of *PR-10 *sequences from *Prunus armeniaca *(AF020784), *P. avium *(U66076), *Pyrus communis *(AF057030), and *Malus domestica *(X83672, Z72425, Z72427). To date, these are the closest non-Fagales relatives of the PR-10 family from *B. pendula*. We confirmed the phylogenetic analysis by constructing a neighbour-joining tree with Kimura two-parameter distances. Bootstrapping was carried out with 1,000 replicates. The results were similar for both analyses; therefore, the results from the neighbor-joining analysis are not shown.

Based on variation among *Mal d 1 *alleles of a single locus compared to alleles of different loci [18], we predefined a cut-off level of 98% identity in order to identify clusters that encompass alleles of the same gene. A limitation of the applied method is that genes that are homozygous and differ less than 98% from each other remain undetected.

Recombination was detected and visualized with Phylpro 1.0 [27]. This program computes the correlation coefficient of pair wise distances between the target sequence and all other sequences on both sides of a sequence position. We used a sliding window of 40 base pairs.

### Isoforms and allergenicity

Nucleotide sequences were aligned codon-by-codon. We analyzed general selection patterns at the molecular level using DnaSp 4.00 [21]. The number of synonymous (K_s_) and non-synonymous substitutions (K_a_) per site were calculated from pair wise comparisons with incorporation of the Jukes-Cantor correction. Nucleotide data were translated into amino acids. Putative isoforms were analyzed for their potential allergenicity by screening those amino acid positions that have been identified as influencing the IgE binding [12,14] and T-cell activation [22].

## Authors' contributions

LJWJG and MJMS designed the study. MJMS, GDE, and MFS designed the experiments. MFS and GDE cloned and sequenced the genes. MFS carried out the sequence analysis. MFS, LJWJG and MJMS drafted the paper

## Figures and Tables

**Table 1:** Cloned and sequenced PR-10 sequences from *B. pendula*. Overview of the individual clones of PR-10 sequences from *B. pendula *'Schneverdinger Goldbirke', 'Tristis', and 'Long Trunk'. Different primers (BpI, BpII) were used. De number of cycles varied between 22 and 30. Confirmed sequences are found in multiple independent PCRs. Unique sequences differ at least by three base pairs from any other sequence from the same cultivar. The remaining sequences are either recombination artifacts or presumably result from base mis-incorporations. The number of alleles included for further analysis is also indicated.

**Cultivar 'Long Trunk'**							
Primer combination no. of PCR cycles	BpI 22 cycles		BpII 24 cycles		BpI 30 cycles		No. of different alleles included

Confirmed sequences	43	(86%)	50	(69%)	24	(45%)	20
Unique sequences	2	(4%)	5	(7%)	5	(9%)	8

Recombination artifacts	1	(2%)	8	(11%)	18^*1^	(34%)	
Base mis-incorporation artifacts	4	(8%)	9	(13%)	6	(11%)	

Total no. of clones	50		72		53		28

**Cultivar 'Tristis'**							

Primer combination no. of PCR cycles	BpI 22 cycles		BpII 24 cycles		BpI 30 cycles		No. of different alleles included

Confirmed sequences	17	(77%)	20	(53%)	26	(54%)	11
Unique sequences	1	(5%)	5	(13%)	0	-	4

Recombination artifacts	2	(9%)	2	(5%)	22^*2^	(46%)	
Base mis-incorporation artifacts	2	(9%)	11	(29%)	0	-	

Total no. of clones	22		38		48		15

**Cultivar 'Schneverdinger Goldbirke'**							

Primer combination no. of PCR cycles	BpI 26 cycles		BpII 26 cycles		BpI 30 cycles		No. of different alleles included

Confirmed sequences	21	(53%)	13	(43%)	21	(43%)	10
Unique sequences	0	-	3	(10%)	3	(6%)	4

Recombination artifacts	12	(30%)	8	(27%)	15	(29%)	
Base mis-incorporation artifacts	7	(18%)	6	(20%)	11	(22%)	

Total no. of clones	40		30		51		14

^*1 ^one sequence had both a recombination and a base mis-incorporation artifact

^*2 ^two sequences had both a recombination and a base mis-incorporation artifact

**Table 2:** Classification and nomenclature of *B. pendula *PR-10 sequences from our cultivars and GenBank. Indicated are the subfamily (I to V), gene designations (PR-10.01A to PR-10.05), allergen designation if the genes are known to be pollen expressed (Bet v 1.01A to Bet v 1.02C), and allele names as defined in Figure 2. Known isoforms [19] are shown, followed, between brackets, by the GenBank accession number. The tissue of origin is shown in case of mRNA-derived GenBank sequences (L = leaves, R = roots, P = pollen).

Sub-family	Gene	Allergen	Allele (GenBank no.)^*1^	Known isoforms (GenBank no.)^*1^	Location of transcription	Sequence identity to reference sequence^*2^
I	PR-10.01A	Bet v 1.01A	Bet v 1.01A01.01^*3 ^(DQ296566)	Bet v 1a = Bet v 1.0101 (X15877), - (AJ001553)	P, P	100%
			Bet v 1.01A02.01 (DQ296567)		-	99.1%
			Bet v 1.01A03.01 (DQ296568)		-	99.1%
				Bet v 1.1501 (Z72429)	-	99.8%
				Bet v 1.1502 (Z72432)	-	99.8%
				Bet v 1.0102 (Z80098)	P	99.8%
				Bet v 1.0103 (Z80099)	P	99.8%
				Bet v 1.2501 (Z80101)	P	98.8%
				Bet v 1.2801 (Z80104)	P	99.5%
				Bet v 1.3001 (Z80106)	P	99.8%
				- (AF124838)	-	98.6%
				- (AJ002107)	P	99.8%
				- (AJ002109)	P	99.3%
				- (AJ006905)	P	99.8% ^*4^
				- (AJ006911)	P	98.6%
				- (AJ006913)	P	99.5% ^*4^
	
	PR-10.01B	Bet v 1.01B	Bet v 1.01B01.01 (DQ296569)	- (AJ001552), - (AJ002106)	P, P	100%
				Bet v 1d = Bet v 1.0401 (X77266)	P	99.3%
				Bet v 1h = Bet v 1.0402 (X77270)	P	99.8%
				- (AJ001551)	P	99.8%
	
	PR-10.01C	Bet v 1.01C	Bet v 1.01C01.01 (DQ296570)		-	100%
			Bet v 1.01C02.01 (DQ296571)		-	99.8%
				Bet v 1f = Bet v 1.0601 (X77268)	P	99.8%
				Bet v 1i = Bet v 1.0602 (X77274)	P	99.8%
				- (AJ001557)	P	99.5%
	
	PR-10.01D	Bet v 1.01D	Bet v 1.01D01.01 (DQ296572)	- (AJ001555)	P	100%
			Bet v 1.01D02.01 (DQ296573)		-	99.1%
				Bet v 1.1701 (Z72430)	-	98.4%
				- (AF124839)	-	98.4%

II	PR-10.02A	Bet v 1.02A	Bet v 1.02A01.01 (DQ296574)		-	100%
			Bet v 1.02A02.01 (DQ296575)		-	99.8%
				- (AJ001554)	P	99.8%
				- (AJ001556)	P	99.8%
	
	PR-10.02B	Bet v 1.02B	Bet v 1.02B01.01 (DQ296576)		-	100%
			Bet v 1.02B02.01a^*5 ^(DQ296577)		-	99.3%
			Bet v 1.02B02.01b (DQ296578)		-	99.3%
				Bet v 1.1801 (Z72431)	-	99.1%
	
	PR-10.02C	Bet v 1.02C	Bet v 1.02C02.01 (DQ296580)	Bet v 1k = Bet v 1.0901 (X77272)	P	100%
			Bet v 1.02C02.02 (DQ296581)		-	99.8%
			Bet v 1.02C01.01 (DQ296579)		-	99.8%
				Bet v 1.20101 (Z72434)	-	99.3%
				Bet v 1c = Bet v 1.0301 (X77265)	P	99.8%
				Bet v 1.1901 (Z72433)	-	98.6%
				Bet v 1m = Bet v 1.1401 (X81972)	P	99.3%
				Bet v 1n = Bet v 1.1402 (X82028)	P	99.3%

III	PR-10.03A	-	PR-10.03A01.01 (DQ296582)		-	100%
			PR-10.03A02.01 (DQ296583)		-	99.8%
	
	PR-10.03B	-	PR-10.03B01.01 (DQ296584)		-	100%
			PR-10.03B-p01^*5 ^(DQ296586)		-	100%^*4^
	
	PR-10.03B^*6^		PR-10.03B02.01 (DQ296585)		-	96.5%
	
	PR-10.03C	-	PR-10.03C01.01 (DQ296587)	- (AJ289771)	-	100%
			PR-10.03C02.01 (DQ296588)		-	99.5%
			PR-10.03C02.02 (DQ296589)		-	99.3%
				Bet v 1.1201 (X77600)	L, R	99.5%
	
	PR-10.03D	-	PR-10.03D01.01 (DQ296590)		-	100%
			PR-10.03D02.01 (DQ296591)		-	99.8%
				Bet v 1.1101 (X77599)	L, R	99.5%

IV	PR-10.04	-	PR-10.0401.01 (DQ296592)		-	100%
			PR-10.0402.01 (DQ296593)		-	98.9%

V	PR-10.05	-	PR-10.0501.03 (DQ296596)	Bet v1 1.1301 (X77601), - (AJ289770)	L, R	100%
			PR-10.0501.02 (DQ296595)		-	99.5%
			PR-10.0501.01 (DQ296594)		-	99.8%
			PR-10.0501.04 (DQ296597)		-	99.8%
			PR-10.0502.01 (DQ296598)		-	99.8%

^*1 ^The known mRNA-derived GenBank sequences contain no intron, while the new gDNA sequences do, aiding in the gene identification.

^*2 ^The upper most allele was taken as a reference sequence, identities are calculated for an aligned stretch of 425 bp from base 28 to 452 of the consensus.

^*3 ^The last two numerals indicate silent mutations (see Results section for further explanation of the nomenclature)

^*4 ^These sequences contain an indel; Sequence identity is calculated excluding the indel.

^*5 ^Pseudogene allele.

^*6^*In vivo *recombination.

## References

[B1] Breiteneder H, Ebner C (2000). Molecular and biochemical classification of plant-derived food allergens. J Allergy Clin Immunol.

[B2] Breiteneder H, Pettenburger K, Bito A, Valenta R, Kraft D, Rumpold H, Scheiner O, Breitenbach M (1989). The gene coding for the major birch pollen allergen BetvI, is highly homologous to a pea disease resistance response gene. EMBO Journal.

[B3] Jarolim E, Rumpold H, Endler AT, Ebner H, Breitenbach M, Scheiner O, Kraft D (1989). IgE and IgG antibodies of patients with allergy to birch pollen as tools to define the allergen profile of Betula verrucosa. Allergy.

[B4] Niederberger V, Pauli G, Gronlund H, Froschl R, Rumpold H, Kraft D, Valenta R, Spitzauer S (1998). Recombinant birch pollen allergens (rBet v 1 and rBet v 2) contain most of the IgE epitopes present in birch, alder, hornbeam, hazel, and oak pollen: A quantitative IgE inhibition study with sera from different populations. J Allergy Clin Immunol.

[B5] Liu JJ, Ekramoddoullah AKM (2004). Characterization, expression and evolution of two novel subfamilies of Pinus monticola cDNAs encoding pathogenesis-related (PR)-10 proteins. Tree Physiology.

[B6] Lüttkopf D, Müller U, Skov PS, Ballmer-Weber BK, Wüthrich B, Hansen KS, Poulsen LK, Kästner M, Haustein D, Vieths S (2002). Comparison of four variants of a major allergen in hazelnut (Corylus avellana) Cor a 1.04 with the major hazel pollen allergen Cor a 1.01. Molecular Immunology.

[B7] Wang CS, Huang JC, Hu JH (1999). Characterization of two subclasses of PR-10 transcripts in lily anthers and induction of their genes through separate signal transduction pathways. Plant Molecular Biology.

[B8] Bohle B, Radakovics A, Jahn-Schmid B, Hoffmann-Sommergruber K, Fischer GF, Ebner C (2003). Bet v 1, the major birch pollen allergen, initiates sensitization to Api g 1, the major allergen in celery: evidence at the T cell level. European Journal of Immunology.

[B9] Neudecker P, Lehmann K, Nerkamp J, Haase T, Wangorsch A, Fötisch K, Hoffmann S, Rösch P, Vieths S, Scheurer S (2003). Mutational epitope analysis of Pru av 1 and Api g 1, the major allergens of cherry (Prunus avium) and celery (Apium graveolens): correlating IgE reactivity with three-dimensional structure. Biochemical Journal.

[B10] Friedl-Hajek R, Radauer C, O'Riordain G, Hoffmann-Sommergruber K, Leberl K, Scheiner O, Breiteneder H (1999). New Bet v 1 isoforms including a naturally occurring truncated form of the protein derived from Austrian birch pollen. Molecular Immunology.

[B11] Hoffmann-Sommergruber K, Vanek-Krebitz M, Radauer C, Wen J, Ferreira F, Scheiner O, Breiteneder H (1997). Genomic characterization of members of the Bet v 1 family: genes coding for allergens and pathogenesis-related proteins share intron positions. Gene.

[B12] Son DY, Scheurer S, Hoffmann A, Haustein D, Vieths S (1999). Pollen-related food allergy: cloning and immunological analysis of isoforms and mutants of Mal d 1, the major apple allergen, and Bet v 1, the major birch pollen allergen. European Journal of Nutrition.

[B13] Swoboda I, Jilek A, Ferreira F, Engel E, Hoffmann-Sommergruber K, Scheiner O, Kraft D, Breiteneder H, Pittenauer E, Schmid E, Vicente O, Heberle-Bors E, Ahorn H, Breitenbach M (1995). Isoforms of Bet v 1, the major birch pollen allergen, analyzed by liquid-chromatography, mass-spectrometry, and cDNA cloning. Journal of Biological Chemistry.

[B14] Ferreira F, Hirtenlehner K, Jilek A, Godnik-Cvar J, Breiteneder H, Grimm R, Hoffmann-Sommergruber K, Scheiner O, Kraft D, Breitenbach M, Rheinberger HJ, Ebner C (1996). Dissection of immunoglobulin E and T lymphocyte reactivity of isoforms of the major birch pollen allergen Bet v 1: Potential use of hypoallergenic isoforms for immunotherapy. Journal of Experimental Medicine.

[B15] Swoboda I, Scheiner O, Heberle-Bors E, Vicente O (1995). cDNA cloning and characterization of three genes in the Bet v 1 gene family that encode pathogenesis-related proteins. Plant Cell and Environment.

[B16] Poupard P, Strullu DG, Simoneau P (1998). Two members of the Bet v 1 gene family encoding birch pathogenesis-related proteins display different patterns of root expression and wound-inducibility. Aust J Plant Physiol.

[B17] Wen J, Vanek-Krebitz M, Hoffmann-Sommergruber K, Scheiner O, Breiteneder H (1997). The potential of Betv1 homologues, a nuclear multigene family, as phylogenetic markers in flowering plants. Mol Phylogenet Evol.

[B18] Gao ZS, van de Weg WE, Schaart JG, Schouten HJ, Tran DH, Kodde LP, van der Meer IM, van der Geest AH, Kodde J, Breiteneder H, Hoffmann-Sommergruber K, Bosch D, Gilissen LJ (2005). Genomic cloning and linkage mapping of the Mal d 1 (PR-10) gene family in apple (Malus domestica). Theor Appl Genet.

[B19] Allergen Nomenclature. http://www.allergen.org/.

[B20] Gao ZS, Weg WE, Schaart JG, Arkel G, Breiteneder H, Hoffmann-Sommergruber K, Gilissen LJ (2005). Genomic characterization and linkage mapping of the apple allergen genes Mal d 2 (thaumatin-like protein) and Mal d 4 (profilin). Theor Appl Genet.

[B21] Rozas J, Sanchez-DelBarrio JC, Messeguer X, Rozas R (2003). DnaSP, DNA polymorphism analyses by the coalescent and other methods. Bioinformatics.

[B22] Jahn-Schmid B, Radakovics A, Luttkopf D, Scheurer S, Vieths S, Ebner C, Bohle B (2005). Bet v 1142-156 is the dominant T-cell epitope of the major birch pollen allergen and important for cross-reactivity with Bet v 1-related food allergens. J Allergy Clin Immunol.

[B23] Whinnett A, Mundy NI (2003). Isolation of novel olfactory receptor genes in marmosets (Callithrix): insights into pseudogene formation and evidence for functional degeneracy in non-human primates. Gene.

[B24] Zylstra P, Rothenfluh HS, Weiller GF, Blanden RV, Steele EJ (1998). PCR amplification of murine immunoglobulin germline V genes: Strategies for minimization of recombination artefacts. Immunology and Cell Biology.

[B25] Judo MSB, Wedel AB, Wilson C (1998). Stimulation and suppression of PCR-mediated recombination. Nucleic Acids Research.

[B26] Odelberg SJ, Weiss RB, Hata A, White R (1995). Template-switching during DNA synthesis by Thermus aquaticus DNA polymerase I. Nucleic Acids Research.

[B27] Weiller GF (1998). Phylogenetic profiles: A graphical method for detecting genetic recombinations in homologous sequences. Mol Biol Evol.

[B28] Wang GCY, Wang Y (1996). The frequency of chimeric molecules as a consequence of PCR co-amplification of 16S rRNA genes from different bacterial species. Microbiology.

[B29] Cline J, Braman JC, Hogrefe HH (1996). PCR fidelity of Pfu DNA polymerase and other thermostable DNA polymerases. Nucleic Acids Research.

[B30] Acinas SG, Sarma-Rupavtarm R, Klepac-Ceraj V, Polz MF (2005). PCR-induced sequence artifacts and bias: Insights from comparison of two 16s rRNA clone libraries constructed from the same sample. Applied and Environmental Microbiology.

[B31] Ohta T (2000). Evolution of gene families. Gene.

[B32] Nei M, Rooney AP (2005). Concerted and birth-and-death evolution of multigene families. Annual Review of Genetics.

[B33] Bolhaar STHP, Van de Weg WE, Van Ree R, Gonzalez-Macebo E, Zuidmeer L, Bruijnzeel-Koomen CAFM, Fernandez-Rivas M, Jansen J, Hoffmann-Sommergruber K, Knulst AC, Gilissen LJWJ (2005). In vivo assessment with prick-to-prick testing and double-blind, placebo-controlled food challenge of allergenicity of apple cultivars. J Allergy Clin Immunol.

[B34] Castro AJ, de Dios Alche J, Cuevas J, Romero PJ, Alche V, Rodriguez-Garcia MI (2003). Pollen from different olive tree cultivars contains varying amounts of the major allergen ole e 1. Int Arch Allergy Immunol.

[B35] The international Populus genome consortium. http://www.ornl.gov/sci/ipgc.

[B36] Gilissen LJ, Bolhaar ST, Matos CI, Rouwendal GJ, Boone MJ, Krens FA, Zuidmeer L, van Leeuwen A, Akkerdaas J, Hoffmann-Sommergruber K, Knulst AC, Bosch D, van de Weg WE, van Ree R (2005). Silencing the major apple allergen Mal d 1 by using the RNA interference approach. J Allergy Clin Immunol.

[B37] Markovic-Housley Z, Degano M, Lamba D, von Roepenack-Lahaye E, Clemens S, Susani M, Ferreira F, Scheiner O, Breiteneder H (2003). Crystal structure of a hypoallergenic isoform of the major birch pollen allergen Bet v 1 and its likely biological function as a plant steroid carrier. Journal of Molecular Biology.

[B38] Thompson JD, Higgins DG, Gibson TJ (1994). Clustal-W - Improving the Sensitivity of Progressive Multiple Sequence Alignment through Sequence Weighting, Position-Specific Gap Penalties and Weight Matrix Choice. Nucleic Acids Research.

[B39] Huelsenbeck JP, Larget B, Miller RE, Ronquist F (2002). Potential applications and pitfalls of Bayesian inference of phylogeny. Systematic Biology.

